# Evaluation of Stereopsis Performance, Gaze Direction and Pupil Diameter in Post-COVID Syndrome Using Machine Learning

**DOI:** 10.3390/biomedicines13112828

**Published:** 2025-11-20

**Authors:** Thomas S. Knauer, Christian Y. Mardin, Jürgen Rech, Georg Michelson, Andreas Stog, Julia Zott, Fritz Steußloff, Moritz Güttes, Helena Sarmiento, Miriam Ilgner, Marie Jakobi, Bettina Hohberger, Julia Schottenhamml

**Affiliations:** 1 Department of Ophthalmology, Universitätsklinikum, Friedrich-Alexander-University of Erlangen-Nürnberg, 91054 Erlangen, Germany; 2 Department of Internal Medicine 3–Rheumatology and Immunology, Deutsches Zentrum Immuntherapie and Center for Rare Diseases Erlangen, Universitätsklinikum Erlangen, Friedrich-Alexander-Universität Erlangen-Nürnberg, 91054 Erlangen, Germany; 3 Talkingeyes & More, Erlangen, 91052 Erlangen, Germany

**Keywords:** Post-COVID, VR-OTS, long COVID

## Abstract

**Background/Objectives**: Post-COVID syndrome (PCS) encompasses symptoms that persist for at least 12 weeks after the onset of a COVID-19 infection and cannot be explained by other causes. The most common symptoms are fatigue, cognitive impairments, and physical limitations. The objective diagnosis of PCS is still challenging, as specific biomarkers are lacking. One possibility to measure cognitive impairment is the virtual-reality-oculomotor-test-system (VR-OTS, Talkingeyes & More, Germany). It shows stereoscopic stimuli in a VR-environment to the test person. While working on the visual tasks, many features are recorded. These features can be categorized into three groups: stereopsis performance, gaze direction, and pupil diameter. The aim of this study was to investigate which of these three feature groups is best to distinguish patients with PCS from a healthy control group. **Methods:** In total, 429 patients with PCS were recruited within the disCOVer 1.0 and disCOVer 2.0 study at the Department of Ophthalmology, Universitätsklinikum (Erlangen, Germany). All patients received VR-OTS measurements. From these measurements, a total of 95 features were extracted, which can be categorized into three groups: gaze direction, pupil diameter, and stereopsis performance. In the first step, support vector machines (SVMs) were trained on these different feature sets and evaluated using the area under receiver operating characteristic (AUROC) as the evaluation metric. In the second step, the same procedure was repeated with each feature independently to investigate which were most the predictive per group. **Results:** The SVM using the pupil diameter features yielded an AUROC of 0.73, the one using the gaze direction features resulted in an AUROC of 0.68. and the stereopsis performance features produced an AUROC of 0.66. The SVM using all VR-OTS data showed an AUROC of 0.68. For the single features, the index of pupillary activity (IPA) showed the best discrimination. Moreover, all features that were evaluated at different difficulties showed the same pattern—that the more difficult test proved to be more predictive. **Conclusions:** The study showed that VR-OTS can distinguish between patients with PCS and healthy control probands. Since different features showed a better performance than others, it makes sense for further studies to use a subset of the available features for further analysis.

## 1. Introduction

In December 2019, the severe acute respiratory syndrome coronavirus 2 (SARS-CoV-2) was observed in Wuhan, Hubei province, China. The virus is transmitted by inhalation or contact with infected droplets. It rapidly spread and caused a global pandemic [1,2]. According to the WHO, more than 778 million cases and over 7 million deaths caused by SARS-CoV-2 had been reported by August 2025 [3]. Even though the infection rate and mortality of SARS-CoV-2 have decreased since the outbreak in 2019, the long disease progression and the limitations in daily activities of patients with Post-COVID syndrome (PCS) makes COVID-19 still a long-lasting societal problem [4,5,6]. PCS is considered a diagnosis by exclusion. Therefore, various diagnostic procedures, including tests like blood samples and pulmonary function tests, are conducted to eliminate other possible medical causes. Since there is no distinct diagnosis for PCS, it is desirable to find objective biomarkers.

Because COVID-19 affects multiple organs, various manifestations can occur, such as pulmonary, renal, hematologic, cardiovascular neuropsychiatric, endocrinal, gastrointestinal, and dermatologic symptoms. [7,8]. A significant number of people suffer from prolonged symptoms after the initial acute phase. [6,9]. If this lasts more than 12 weeks, it is defined as Long COVID(LC)/post-COVID-19-syndrome (PCS)/post-acute sequelae of COVID-19 (PASC) by the WHO [10,11,12]. In most cases, the symptoms are fatigue, aches and pains in the muscles, feeling breathless, headaches, alterations in taste, and cognitive impairment [12].

One method to measure cognitive impairment is the virtual-reality-oculomotor-test-system (VR-OTS, Talkingeyes & More, Germany). The device generates tasks using a virtual 3D environment. During task execution, several of the participant’s biomarkers are measured. These biomarkers can be categorized into three groups: stereopsis performance, pupil diameter, and the gaze direction. Mehringer et al. [13] performed a study using the VR-OTS to distinguish patients with PCS from a healthy cohort. However, they only used 73 features that were provided by the device. All three groups of features (stereopsis performance, gaze direction, and pupil diameter) showed conspicuous values for patients with PCS. However, the disadvantage of the study by Mehringer et al. [13] was the very small patient cohort. Güttes et al. [14] also used the VR-OTS device to measure patients with PCS, and showed significantly prolonged reaction times and worse accuracy for patients with PCS. There are some studies investigating similar processes with a simpler setup, using a stimulus on a 2D-screen in combination with a mobile pupil or gaze tracker. Schmidt et al. [15] documented both a prolonged reaction time and smaller pupils with higher oscillations for patients with PCS. Birirgen et al. [16] demonstrated a lower latency and a shorter duration of the pupil response of COVID-19 patients, while Yurtaser et al. [17] revealed a smaller pupil with lower speed. Garcia et al. [18] showed alterations in the eye movement of patients with PCS [18].

Since the aforementioned studies showed that VR-OTS has great potential for measuring cognitive impairment and distinguishing patients with PCS from healthy controls, pupil and gaze alterations detected by VR-OTS could prove to be valuable biomarkers for PCS. However, VR-OTS records many features. So, the goal of this study was to extract 95 features from the VR-OTS measurements and evaluate which of them performed best. To do so, the different VR-OTS features were used as input to support vector machines (SVMs) and the area under receiver operator characteristic (AUROC) was computed to evaluate their performance. In the first step, the parameters were categorized into three groups (gaze data, pupil diameter, and stereopsis performance). Each of these groups was used as input to the SVM. Moreover, an overall model was trained including all features. In the second step, each parameter was evaluated on its own to see which features are most predictive.

## 2. Materials and Methods

### 2.1. Study Population

A total of 429 participants (242 female, 187 male, mean age 39.7 ± 12.7) were recruited at the Department of Ophthalmology, Universitätsklinikum Erlangen, Friedrich-Alexander Universität Erlangen-Nürnberg, Erlangen, Germany, during the course of the disCOVer1.0- and disCOVer2.0-study: 330 patients with PCS (193 female, 137 male, mean age 40.9) and 99 healthy controls (female 49, male 50, mean age 35.7). The demographic data are given in Table 1. For inclusion, patients of the PCS group had to prove the infection by a Polymerase Chain Reaction (PCR) Test and had to have suffered from symptoms for at least 3 months after acute infection according to the German guidelines [10]. Exclusion criteria were an age below 18 years, pre-existing ocular disorders, or systemic disorders with possible ocular involvement and a visual acuity below 0.8 on the weaker eye. Informed written consent was obtained from all participants. The study was reviewed and approved by the ethics committee of the Friedrich-Alexander Universität Erlangen-Nürnberg (295_20_B) and was performed in accordance with the tenets of the Declaration of Helsinki.

### 2.2. VR-OTS

**Figure 1:**
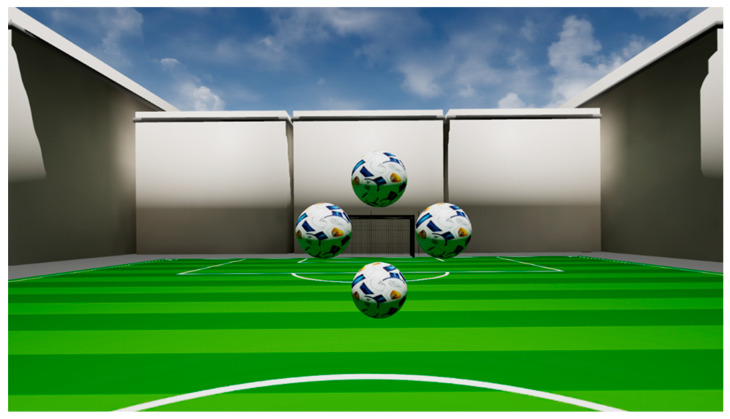
Visual depiction of the visual stimulus presented to a participant: Four balls (central position) are rendered in a rhomboid arrangement. The patient has to detect which of the balls is rendered closer.

The virtual-reality-oculomotor-test-system (VR-OTS, Talkingeyes & More, Erlangen, Germany) is a device to analyze ocular function and cognitive impairment. It uses the HTC Vive Pro Eye (HTC Corporation, Taoyuan, Taiwan) to create a virtual 3D-environment. Four balls in a rhomboid arrangement are presented at a virtual distance of 200 cm in front of the patient. The set of the four balls can occur in nine possible positions (central, down, lower right, right, upper right, up, upper left, left, lower left). The arrangement of the balls is displayed at Figure 1. One of the balls is rendered closer to the patient. The difference in the distance between the ball rendered closer and the remaining balls defines the difficulty of the task. In VR-OTS, the patients are presented with tasks of three different difficulty levels (disparities of 250, 550, 1100 arc-seconds (arcsec), where a lower disparity indicates a higher difficulty). The patient must choose the closest of the four balls by pressing the analog key on a keyboard. In each run, all combinations of the three disparities and the nine testing positions are displayed three times in a random order. This makes a total of 81 stereoscopic tasks per run. Each patient performs three trials. On average, the whole procedure takes about 5 min. Only the third run is used for analysis, while the first two serve as practice. In total, 79 features of three data groups were measured, which are explained in more detail in the following.

#### 2.2.1. Stereopsis Performance Features

One data group was about the stereopsis performance of the patient. Reaction time and the number of correct responses were recorded. Beside the accuracy, which is the percentage of correctly given responses, 9 statistical parameters (maximum, minimum, median, mean, standard deviation, variance, skewness, kurtosis) of the reaction time were calculated for each difficulty. For the reaction time, only the correctly identified balls were evaluated. Moreover, the gain, which is defined as the difference between the mean reaction time of two difficulties, was computed for the disparities 275 and 550 and 275 and 1100 [19]. This led to a total of 29 features in the stereopsis performance group.

#### 2.2.2. Pupil Diameter Features

The second data group was about pupil diameter, which was recorded by an eyetracker. The VR-OTS determines the maximum, minimum, range, median, mean, standard deviation, variance, skewness, kurtosis of the pupil diameter for each eye. However, in this study, only the data of the right eye was used, because we assume that there is a correlation between the right and the left eye of the same patient. Moreover, the mean of the index of pupillary activity (IPA) and the mean of the low/high index of pupillary activity (LHIPA) are computed across the entire pupillary signal. IPA [20] and LHIPA [21] are indices to measure cognitive load based on the oscillation and variation in pupil diameter signal. Additionally, values for the signal slopes are calculated. These signal slopes are the linear regression of the pupil diameter for every stimulus. Because the pupil reaction to a stimulus is delayed, every slope is divided into two subsections. The first one is mainly influenced by the prior stimulus. The second one shows the reaction to the current stimulus [22]. IPA, LHIPA, and the slope values were calculated for every disparity. This led to a total of 26 features.

#### 2.2.3. Gaze Behavior

The VR-OTS can also document the distance of gaze direction changes via an eye tracker. From these changes, the angular velocity can be computed using the asymptotic model described by Duchowski et al. [23]. From the gaze direction change and angular velocity, the maximum, median, mean, standard deviation, variance, skewness, kurtosis, and entropy were calculated. Moreover, the number of fixations and fixations per trial were counted. Also, the time every target (background, each ball) got fixated was recorded. This led to a total of 24 values about gaze directions and fixation.

#### 2.2.4. Evaluation

For the evaluation, the available data was split into a 60% training, 20% validation, and 20% test set. In the first step, the features of the three groups (stereopsis performance, gaze direction, and pupil diameter) and all acquired features were used as input to train multiple support vector machines (SVMs) with different architectures on the training set. All of them were subsequently evaluated on the validation set using the area under receiver operating characteristics (AUROC) as the evaluation metric. A 10-fold cross-validation was performed. For each of these four groups, the SVM architecture that achieved the highest mean AUROC on the validation set was chosen for the final evaluation on the test set. In a second step, the same procedure was repeated for each feature individually to evaluate which of them showed the best performance per group. 

The programming was done in python (version 3.9) using the scikit-learn library (version 1.3.2).

## 3. Results

All three feature groups (stereopsis performance, the pupil diameter, and the gaze direction) showed a mean AUROC above 0.66 and are consequently suitable methods to distinguish patients with PCS from healthy controls. The best discrimination was achieved by the pupil diameter, which had a mean AUROC of 0.73, followed by gaze direction and stereopsis performance. The mean AUROC values are shown in detail in Table 2.

The stereopsis feature group showed the lowest AUROC of 0.66. For the single features of this group, the minimum reaction time at the hardest difficulty level (disparity 275) yielded the best AUROC of 0.68. The best mean AUROC values of single features in this group was achieved by the mean (mean 275: 0.68; mean 550: 0.67; mean 1100: 0.67) and median (median 275: 0.68; median 550: 0.68; median 1100: 0.66) of the reaction time, while accuracy was less predictive (accuracy 275: 0.59; accuracy 550: 0.56; accuracy 1100: 0.53). For the mean and median of reaction time and accuracy, mean AUROC values decreased along with difficulty.

The gaze direction feature group achieved an AUROC of 0.68. The best feature in this group was the fixation duration, which had an AUROC of 0.70. The fixation time on the background also showed predictive value (0.67) and exceeded the fixation time on the four balls (right ball: 0.65; top ball: 0.61; left ball: 0.62; bottom ball: 0.64). The maximum of angular velocity and the amplitude of the gaze direction yielded the worst AUROC values of 0.52 and 0.54 in this group.

Pupil diameter was the best performing group of parameters, with an AUROC of 0.73. The values for mean IPA (IPA 275: 0.70; IPA 550: 0.71; IPA 1100: 0.66) and mean LHIPA (LHIPA 275: 0.67; LHIPA 550: 0.67; LHIPA 1100: 0.65) of the individual difficulties performed best. The minimum of the pupil diameter at the hardest difficulty yielded an AUROC of 0.65. The worst mean AUROC values in this group were achieved by the slope features for any difficulty, with mean AUROC-values between 0.48 (Slope 2 550) and 0.58 (Slope 275).

The mean AUROC values and their standard deviations for all single features can be found in Table A1, Table A2 and Table A3 in the Appendix A.

## 4. Discussion

PCS is defined as symptoms persisting more than 12 weeks after an acute COVID-19 infection. Most patients report neurological symptoms such as significantly impaired thinking or concentration problems [7,12,24]. 

These neurological symptoms could be explained by neurotropism in COVID-19, which is examined in multiple studies [25]. While Voruz et al. [26] conclude that the central nervous system (CNS) is affected by SARS-CoV-2, Whu et al. [27] describe involvement of cerebrospinal fluid and the brain. This CNS damage can lead to alterations in pupil response [16,17] and eye movement [18,28]. In earlier investigations, stereoscopic visual tasks were used to quantify traumatic brain injuries [29]. We assume that this can also be applied to measure cognitive impairment in patients with PCS.

The VR-OTS is a functional test system that can examine this cognitive impairment. The device shows 4 balls in a 3D-enviroment. The participant must identify one ball, which is projected closer to the patient than the remaining three balls, while measurements are performed. A total of 95 features are extracted from the measurement and can be categorized into three groups: stereopsis performance, gaze direction, and pupil diameter. Since these are many features, the aim of this study was to examine which of them is best to discriminate PCS-patients from healthy controls to simplify diagnosis in future.

Similar approaches have been described in the literature: Cena et al. [18] and Duan et al. [30] analyzed the gaze behavior of patients with PCS fixating on a 2D-screen. Vinela-Navarro et al. extended the experimental setup by incorporating a pupil tracker [31]. In the study of Carbone et al. [32], in addition to observing the gaze behavior on a 2D-screen, a task for measuring the reaction time was added. Maiorana et al. determined the reaction time via a visual detection task on a phone. None of the methods presented measures stereopsis performance, gaze directions, and pupil diameter simultaneously. Moreover, none of them used a 3D stimulus, in contrast to VR-OTS.

In our study, stereopsis performance revealed the worst mean AUROC-value, while in the study of Mehringer et al. [13] this group of features outperformed gaze direction and the pupil diameter. A reason for this might be that Mehringer et al. only included 35 patients (20 patients with PCS and 15 controls) in their study, which may have led to different results compared to our study with 429 patients (330 patients with PCS and 99 controls).

In our study, the minimum reaction time at a disparity of 275 had the best discrimination. Also, the median and mean of the reaction time showed high AUROC values. One observation here was that the AUROC dropped along with the difficulty. This is in line with previous studies. Mehringer et al. [13] showed that the mean and median are increased in patients with PCS. In a further study of Güttes et al. [14], the reaction time of patients with PCS was significantly prolonged, while the *p*-value decreased inverse to the difficulty [14]. Prolonged reaction times in PCS patients were also found in multiple other studies [15,33,34]. An increase in differences with more complex tasks was also reported by Santoyo-Mora et al. [34]. The reason therefore is presumably cognitive deficiencies. Santoyo-Mora et al. [34] attribute this to a higher cognitive performance load, which causes patients with PCS to require more time. This is consistent with our observation that the AUROC values increase along with the difficulty, particularly regarding the minimum reaction time at our highest difficulty level.

The accuracy values for all difficulties of the stereopsis feature group were among the worst predictive parameters. In the case of Güttes et al. [14], accuracy was significantly reduced in patients with PCS, although the level of significance was notably lower than reaction time. However, this study only investigated whether there is a statistically significant difference between patients with PCS and healthy controls; it did not investigate the performance of the features. Mehringer et al. [13] could also show that the accuracy is less predictive than the reaction time. In our study, the accuracy features at higher difficulties proved to be more predictive. Güttes et al. [14] could also show that the statistical significance decreases with lower difficulty. One possible explanation therefore might be that patients with PCS usually do not report worse visual acuity. Consequently, they should be able to correctly identify the closer ball. However, they might have a prolonged cognitive processing time, leading to a higher reaction time. Moreover, in our study, only patients with a visual acuity larger than 0.8 were included, which can also eliminate further differences in the accuracy.

The AUROC value of the gaze direction feature group performs better in our study than the stereopsis group but worse than the pupil diameter group. From this group, the fixation duration emerges as the most predictive parameter, which was evident in all possible fixation directions. This is consistent with the results of Mehringer et al. [13]. According to the author, the main reason might be that patients with PCS take longer to complete the entire procedure, just like the reaction times in the stereopsis performance feature group. The fixation on the background was the second-best performing feature. We suspect that a possible cause for this might be a prolonged fixation on the background caused by a high cognitive load, as described by Martinez-Cedello et al. [35]. The maximum of the angular velocity and the maximum of the amplitude of gaze direction were the worst parameters of the gaze direction feature group. Mehringer et al. [13] observed the same effect.

The pupil diameter feature group showed the best mean AUROC-values in our study and performed better than in the earlier study by Mehringer et al. [13]. This may be again due to the small sample size used in their study. Multiple other studies also observed differences in the pupil behavior in patients with PCS compared to healthy controls [15,16,17]. The AUROC of the pupil diameter feature achieved a value of 0.73, indicating a moderate to good ability to discriminate between healthy patients and patients with PCS [36]. In our study, IPA and LHIPA showed the highest mean AUROC values at every difficulty and that they correlate with difficulty. Since IPA and LHIPA are direct measures of the cognitive load, this is a strong hint that there is a cognitive impairment in patients with PCS. Mehringer et al. [13] found in their study that only LHIPA at the hardest difficulty could distinguish between patients with PCS and healthy controls. A reason for this might again be the small sample size, which could only detect stronger effects. The minimum diameter also seemed to be a good feature for the differentiation of patients with PCS from healthy controls. A smaller peak diameter for patients with a higher cognitive load could be described by [37,38]. Consequently, patients with PCS could show smaller pupil sizes due to their increased cognitive load.

The worst mean AUROC values in the pupil diameter group were generated by the slope features. Mehringer et al. [13] could also not find a substantial difference in these features between patients with PCS and healthy controls. They attribute this to the flexible processing time of each stimulus. To improve these features, the test system would need to be modified to include a fixed time per stimulus [13].

The study is not without limitations. One limitation of the present study is the lack of differentiation between subgroups of patients. It is assumed that different molecular mechanisms such as persisting viral reservoirs or viral reactivation [39], abnormal blood clotting [40], endothelial dysfunction [41], and immune and autoimmune dysregulation [42] can lead to PCS. Consequently, cognitive impairment might not be present in all patients with PCS, which could influence our results. Thus, investigating stereopsis performance, gaze direction, and pupil diameter in different PCS subgroups might increase its diagnostic value. In addition, further stratification can be done by duration of illness, severity of symptoms, type of pathogenesis, or gender. Furthermore, only a European patient population was included. Moreover, the PCS group contained more patients than the control group, in particular more female patients. Also, only 429 patients were included. Consequently, the results might not be generalizable or transferrable to other ethnicities. Richer or semantically engaging targets might increase the attentional engagement and modulate gaze behavior. Therefore, future studies should compare the current minimalistic stereotarget with more visually engaging environments to evaluate whether additional visual richness improves the signal-to-noise ratio without compromising task specificity.

## 5. Conclusions

The study showed that VR-OTS is a useful tool for distinguishing between patients with PCS and a healthy control group. Since different features showed a better performance than others, it would make sense for further studies to use a subset of the available features for further analysis. For example, the IPA values were the best performing, indicating that patients with PCS suffer from cognitive impairment. Furthermore, it was shown that tasks with a higher difficulty leaded to more predictive results.

## Figures and Tables

**Table 1:** Demographic data of patients with PCS and healthy controls.

	PCS (*n* = 330)	Control (*n* = 99)
Sex (male/female)	137 (41.5%)/193 (58.5%)	50 (50.5%)/49 (49.5%)
Age	40.9 (±11.9)	35.7 (±14.5)

**Table 2:** Mean AUROC values and their standard deviation of stereopsis performance, pupil diameter, gaze direction and all VR-OTS features.

Parameter Group	Mean AUROC ± Standard Deviation
pupil diameter	0.73 ± 0.09
gaze direction	0.68 ± 0.07
stereopsis performance	0.66 ± 0.09
all extracted VR-OTS	0.71 ± 0.07

## Data Availability

The dataset analyzed during the current study is not publicly available due to general data privacy regulations but is available from the corresponding author.

## Abbreviations

The following abbreviations are used in this manuscript:

arcsec	arc-sec
AUROC	area under receiver operating characteristic
CNS	Central nervous system
IPA	Index of Pupillary Activity
LC	long COVID
LHIPA	Low/High Index of Pupillary Activity
PASC	post-acute sequelae of COVID-19
PCS	Post-COVID-19-syndrome
PCR	Polymerase Chain Reaction
SARS-CoV-2	severe acute respiratory syndrome coronavirus 2
SVM	Support vector machines
VR-OTS	Virtual-Reality-oculomotor-test-system

## Appendix A

**Table A1:** Mean AUROC values and their standard deviation of features of the stereopsis performance.

Parameter	Difficulty	Mean AUROC ± Standard Deviation
Accuracy	275	0.59 ± 0.10
maximum (reaction time)	275	0.63 ± 0.13
minimum (reaction time)	275	0.68 ± 0.10
median (reaction time)	275	0.68 ± 0.10
mean (reaction time)	275	0.68 ± 0.09
standard deviation (reaction time)	275	0.64 ± 0.10
variance (reaction time)	275	0.62 ± 0.11
skewness (reaction time)	275	0.62 ± 0.06
kurtosis (reaction time)	275	0.63 ± 0.09
accuracy (reaction time)	550	0.56 ± 0.07
maximum (reaction time)	550	0.68 ± 0.08
minimum (reaction time)	550	0.64 ± 0.09
median (reaction time)	550	0.68 ± 0.08
mean (reaction time)	550	0.67 ± 0.09
standard deviation (reaction time)	550	0.66 ± 0.08
variance (reaction time)	550	0.66 ± 0.07
skewness (reaction time)	550	0.54 ± 0.09
kurtosis (reaction time)	550	0.54 ± 0.12
accuracy (reaction time)	1100	0.53 ± 0.07
maximum (reaction time)	1100	0.64 ± 0.10
minimum (reaction time)	1100	0.65 ± 0.08
median (reaction time)	1100	0.66 ± 0.10
mean (reaction time)	1100	0.67 ± 0.10
standard deviation (reaction time)	1100	0.62 ± 0.10
variance (reaction time)	1100	0.62 ± 0.10
skewness (reaction time)	1100	0.59 ± 0.09
kurtosis (reaction time)	1100	0.60 ± 0.09
median gain Disp 275-1100	275-1100	0.60 ± 0.10
median gain Disp 550-1100	550-1100	0.60 ± 0.07

**Table A2:** Mean AUROC values and their standard deviation of features of the gaze direction.

Parameter	Mean AUROC ± Standard Deviation
maximum (amplitude)	0.54 ± 0.06
median (amplitude)	0.58 ± 0.17
mean (amplitude)	0.61 ± 0.11
Standard deviation (amplitude)	0.59 ± 0.10
variance (amplitude)	0.59 ± 0.10
skewness (amplitude)	0.65 ± 0.08
kurtosis (amplitude)	0.61 ± 0.11
entropy (amplitude)	0.61 ± 0.11
maximum (angular velocity)	0.52 ± 0.07
median (angular velocity)	0.58 ± 0.17
mean (angular velocity)	0.61 ± 0.12
Standard deviation (angular velocity)	0.60 ± 0.11
variance (angular velocity)	0.60 ± 0.11
skewness (angular velocity)	0.62 ± 0.09
kurtosis (angular velocity)	0.62 ± 0.10
entropy (angular velocity)	0.62 ± 0.09
fixation duration	0.70 ± 0.09
fixation duration (background)	0.67 ± 0.08
fixation duration (ball up)	0.61 ± 0.07
fixation duration (ball down)	0.64 ± 0.10
fixation duration (ball left)	0.62 ± 0.10
fixation duration (ball right)	0.65 ± 0.10
ball transitions	0.64 ± 0.10
mean ball transitions per trial	0.64 ± 0.10

**Table A3:** Mean AUROC values and their standard deviation of features of the pupil diameter.

Parameter	Difficulty	Mean AUROC ± Standard Deviation
maximum (diameter)	275-550-1100	0.60 ± 0.09
minimum (diameter)	275-550-1100	0.65 ± 0.09
median (diameter)	275-550-1100	0.63 ± 0.09
mean (diameter)	275-550-1100	0.63 ± 0.09
standard deviation (diameter)	275-550-1100	0.55 ± 0.09
variance (diameter)	275-550-1100	0.55 ± 0.10
skewness (diameter)	275-550-1100	0.48 ± 0.09
kurtosis (diameter)	275-550-1100	0.51 ± 0.10
range (diameter)	275-550-1100	0.51 ± 0.08
mean IPA	275-550-1100	0.53 ± 0.15
mean LHIPA	275-550-1100	0.55 ± 0.08
mean IPA	275	0.70 ± 0.09
mean LHIPA	275	0.67 ± 0.10
mean slope	275	0.58 ± 0.07
mean slope 1	275	0.52 ± 0.11
mean slope 2	275	0.52 ± 0.13
mean IPA	550	0.71 ± 0.14
mean LHIPA	550	0.67 ± 0.05
mean slope	550	0.57 ± 0.08
mean slope 1	550	0.50 ± 0.10
mean slope 2	550	0.48 ± 0.09
mean IPA	1100	0.66 ± 0.09
mean LHIPA	1100	0.65 ± 0.06
mean slope	1100	0.49 ± 0.09
mean slope 1	1100	0.57 ± 0.10
mean slope 2	1100	0.52 ± 0.12
